# Binder Free and Flexible Asymmetric Supercapacitor Exploiting Mn_3_O_4_ and MoS_2_ Nanoflakes on Carbon Fibers

**DOI:** 10.3390/nano10061084

**Published:** 2020-05-31

**Authors:** Amjid Rafique, Usman Zubair, Mara Serrapede, Marco Fontana, Stefano Bianco, Paola Rivolo, Candido F. Pirri, Andrea Lamberti

**Affiliations:** 1 Politecnico di Torino, Dipartimento di Scienza Applicata e Tecnologia (DISAT), Corso Duca Degli Abruzzi, 24, 10129 Turin, Italy; usman.zubair@polito.it (U.Z.); stefano.bianco@polito.it (S.B.); paola.rivolo@polito.it (P.R.); fabrizio.pirri@polito.it (C.F.P.); 2 Faculty of Engineering and Technology, National Textile University, Faisalabad 38000, Pakistan; 3 Istituto Italiano di Tecnologia, Center for Sustainable Future Technologies, Corso Trento, 21, 10129 Turin, Italy; mara.serrapede@iit.it (M.S.); marco.fontana@iit.it (M.F.)

**Keywords:** supercapacitors, Mn_3_O_4_, MoS_2_, fiber-shaped, flexible

## Abstract

Emerging technologies, such as portable electronics, have had a huge impact on societal norms, such as access to real time information. To perform these tasks, portable electronic devices need more and more accessories for the processing and dispensation of the data, resulting in higher demand for energy and power. To overcome this problem, a low cost high-performing flexible fiber shaped asymmetric supercapacitor was fabricated, exploiting 3D-spinel manganese oxide Mn_3_O_4_ as cathode and 2D molybdenum disulfide MoS_2_ as anode. These asymmetric supercapacitors with stretched operating voltage window of 1.8 V exhibit high specific capacitance and energy density, good rate capability and cyclic stability after 3000 cycles, with a capacitance retention of more than 80%. This device has also shown an excellent bending stability at different bending conditions.

## 1. Introduction

In very recent years, supercapacitors (SCs) have gained a considerable attention, because of their ability to deliver higher power density than batteries, and to store higher energy density than electrostatic capacitors [1,2]. To bridge the gap of energy density between batteries and conventional capacitors, supercapacitors have been used in a variety of applications, ranging from portable electronic devices, hybrid electrical vehicles and large industrial scale power and energy management [3,4].

The behavior of SCs is strongly influenced by the layout, sealing and packaging technology, the separator, the electrolyte, the current collectors and, most importantly, it is influenced by the active materials at the electrodes [5]. For this reason, it is essential to develop new materials and new structures able to deliver high specific capacitance, and to sustain high cycling stability with a good rate capability. Based on the charge storage mechanism, SCs are classified as electrical double layer capacitors (EDLCs) and pseudocapacitors. The EDLCs are characterized by extremely large surface area achieved by a good compromise between open pores, size of the pores and dimension of the charges in the electrolyte that generate the double-layer region with the electrode surface. Usually carbon-based materials give rise to large values of EDL capacitance. Together with this phenomenon, a large group of metal oxides, transition metal dichalcogenides, carbides and conducting polymers exhibit pseudocapacitance that is generated by fast and reversible Faradaic reactions occurring at the electrode/electrolyte interface, or in the bulk very close to the electrode surface. Pseudocapacitors show higher capacitance when compared to EDLC-based devices, but they usually have a limited cyclic life, due to the degradation of the active material upon continuous redox reactions. Even though the two phenomena are intrinsically different, they can be grouped because of their intrinsic kinetics, allowing to systematically study the charge storage mechanisms in hybrid electrodes and differentiate capacitive-controlled and diffusion-controlled charge storage mechanisms [6]. The challenge of the current SCs is to increase the energy density without sacrificing power density and cyclic life [7]. According to the equation which describes the energy stored in a capacitor:

E = 1/2CV^2^

where C is the capacitance and V the voltage, there are two possibilities to enhance the energy density of the device: (i) by increasing the specific capacitance of the device, (ii) by increasing the operating potential window. The main limitation to the increase of the voltage window is the stability of the electrolyte, especially when working with aqueous-based solutions, due to the electrolysis of water. This limitation occurs also in the water-based gel-polymer electrolytes, greatly required to realize portable and wearable supercapacitors [8].

The development of asymmetric supercapacitors in which the anode and cathode are clearly distinct is a good approach to improve their energy density, because the combination of appropriate electrode materials with complementary potential windows allows to enlarge the operating voltage of the device [9,10]. Carbon-based materials are usually exploited as anode, while pseudocapacitive materials, such as oxides, are usually set at the cathode. Together with carbon-based materials, transition metal dichalcogenides (WS_2_ [11,12], Bi_2_S_3_ [13,14], Bi_2_Te_3_ [15], MoSe_2_ [16,17], MoTe_2_ [18,19], ZnS [20,21], FeS_2_ [22]) can be used as an anode, thanks to their intrinsic properties. Among them, MoS_2_ has a layered structure similar to graphite and its large surface area, good electrical conductivity (metallic when in 1T phase), fast ionic conductivity and the 2D-like morphology that make it a potential candidate for high-performing electrode [23,24].

On the other hand, manganese dioxide (MnO_2_) is well-known to behave as ideal pseudocapacitor material, due to its fast-redox reactions, and can be used as efficient cathode [25,26,27]. MnO_2_ can be easily electrodeposited onto current collectors by anodic or cathodic electrodeposition [28,29], and this soft-chemical method also makes it possible to conformally coat very rough current collectors. The most common morphology of MnO_2_ achievable by anodic electrodeposition is a porous nanostructured three-dimensional network [28,30,31] or a matrix of interconnected micro- and nano-scale fibers, rods and nanosheets [28,31,32]. Moreover, recently, the pseudocapacitive performance of manganese oxide with oxygen vacancies (MnO_2-x_ have been reported showing better values than bare MnO_2_ [33].

Herein, an asymmetric supercapacitor exploiting carbon fibers (CF) as current collectors, Mn_3_O_4_ as the cathode, MoS_2_ as anode and polyvinyl alcohol (PVA) gel electrolyte is reported. The Mn_3_O_4_ phase is achieved by combining electrodeposition of MnO_x_ on CF and its subsequent reduction by annealing the sample in an atmosphere rich in H_2_. MoS_2_ instead was obtained by hydrothermal synthesis, and then dip coated onto the CF. In both cases, the CFs play an important role, allowing better exploitation of the active material, good material loading and flexibility. The asymmetric supercapacitor exhibits a wide potential window of 1.8 V, a high specific capacitance of 70 F g^−1^, high energy and power density values, together with good cycling stability and bending stability.

## 2. Materials and Methods

### 2.1. Electrodeposition of Manganese Oxide and Annealing to Spinel Mn_3_O_4_

The deposition of MnO_x_ electrodes on carbon fibers was carried out by a simple 2-step galvanostatic electrodeposition in a three electrodes cell, where a Pt sheet and a Ag/AgCl sat. were used as counter and reference electrodes, respectively, as described elsewhere [34]. Before starting the electrodeposition, carbon fibers (Panex^®^35, ZOLTEK) were gently cleaned in an acetone, ethanol and deionized water (1:1:1) ratio, and then blocked onto a microscope glass slide with a copper tape, to improve the electrical contact. The deposition was carried out under a current density of 1 mA/cm^2^ for 45 min and 0.5 mA/cm^2^ for 75 min in a solution containing 0.1 M manganese (II) acetate (Mn (CH3COO)_2_, > (98%), Sigma-Aldrich), as MnO_2_ precursor, and 0.1 M of sodium sulfate (Na_2_SO_4_, 98%, Sigma-Aldrich), as supporting electrolyte. After the first step of 45 min, the electrodes were calcined for 1 h at 150 °C, while after the second step of 75 min deposition, the calcination was carried out at 300 °C for 1 h and a black layer of manganese oxide on fiber-electrodes was obtained with an average mass loading of 0.25 mg cm^−1^. The mass was estimated by weighting the carbon fibers before and after the electrodeposition. The thermal treatment of electrodeposited electrodes was carried out by annealing the fibers under a constant hydrogen flow at 250 °C for 3.5 h (2.5 °C per min ramp applied). After this treatment, the manganese oxide’s stoichiometry changes to Mn_3_O_4_, as shown in the “Results and Discussion” section.

### 2.2. Synthesis of MoS_2_ Flakes and Electrodes Fabrication

The synthesis of MoS_2_ nanoflakes was carried out with a simple hydrothermal method, as described elsewhere [24]. A total of 20 mg of dried flakes of MoS_2_ were sonicated in 10 mL distilled water, and the bundle of fibers was gently dipped into this dispersion and allowed to dry until the desired mass per length was obtained. No addition of binder was employed.

### 2.3. Physical-Chemical Characterization

The crystallographic characterization of the as-synthesized samples was performed with a Panalytical X’Pert MRD Pro in Bragg/Brentano configuration X-ray diffractometer (XRD) with a Cu Kα X-ray source.

Raman spectrum was achieved using Renishaw InVia Reflex micro-Raman spectrometer (Renishaw plc, Wottonunder-Edge, UK), equipped with a cooled CCD camera, with a laser excitation wavelength of 514.5 nm and a laser spot size of 10 μm.

The characterization of morphology was performed with a Field-Emission Scanning Electron Microscope (FESEM Supra 40, manufactured by Zeiss), equipped with an Oxford instruments Si (Li) detector for Energy Dispersive X-ray (EDX) spectroscopy.

X-ray photoelectron spectroscopy (XPS) measurements were performed with a Physical Electronics system (PHI 5000 Versa Probe), equipped with monochromatic X-ray Al Kα (1486.6 eV energy) source and a combined e^-^ and Ar^+^ system for charge compensation. Different pass energy values were adopted for survey spectra (187.85 eV) and high-resolution scans (23.5 eV). The calibration of the binding energy scale was obtained by assigning an energy value of 284.8 eV to the main contribution (C-C) of the C 1s region. Background subtraction in high-resolution scans was performed by means of a Shirley function. Data analysis was carried out with Casa XPS software. Transmission electron microscopy (TEM) analyses were performed on an FEI Tecnai F20 ST transmission electron microscope.

### 2.4. Electrochemical Characterization and Device Assembling

The storage behavior of the individual electrodes, Mn_3_O_4_ and MoS_2_ coated fibers, was studied in a three electrodes cell in 1 M Na_2_SO_4_ electrolyte, purged with nitrogen for 20 min prior to any measurement. Platinum bar and Ag/AgCl sat. KCl were employed as counter and reference electrodes respectively. Cyclic voltammetry at multiple scan rates was carried out between −0.6 V and 0.15 V vs. Ag/AgCl and between 0.1 V and 0.85 V vs. Ag/AgCl for MoS_2_ and Mn_3_O_4_ electrodes, respectively. AC impedance spectroscopy was carried out at 5 mV amplitude between 100 kHz and 10 mHz. In the asymmetric devices the mass or length of the MoS_2_ decorated carbon fibers electrode was tuned, in order to counterbalance the charges stored on the Mn_3_O_4_ electrode and the potential voltage window was determined. An asymmetric device was first characterized in 1M Na_2_SO_4_ electrolyte by means of cyclic voltammetry and chronopotentiometry in 1.8 V voltage window. An asymmetric device was also fabricated in two parallel electrodes configuration using hydrogel electroyte (the gelification of the liquid electrolyte was done by adding PVA (89,000–98,000, 99+% hydrolyzed Sigma Aldrichin 1M Na_2_SO_4_ Sigma Aldrich). The anode (MoS_2_) and cathode (Mn_3_O_4_) were first dipped in hydrogel and left in ambient condition for 30 min for semi-solidification. Then, both the electrodes were put between two kapton^®^ tapes in parallel configuration and sealed. Characterization was also performed for this device by means of cyclic voltammetry, chronopotentiometry in 1.8 V voltage window. Moreover, to appreciate the potentialities of those devices, the capacitance retention was studied at different bending angles. All the experiments were performed with a Metrohm Autolab PGSTAT128 Potentiostat/Galvanostat and Nova 2.1 software.

## 3. Results and Discussion

### 3.1. Characterization of the Material at the Cathode

Binder-free highly porous Mn_3_O_4_ nanoflakes were obtained via a 2-step process, which involved the galvanostatic electrodeposition of the manganese oxide and its surface modification through hydrogenation.

**Figure 1:**
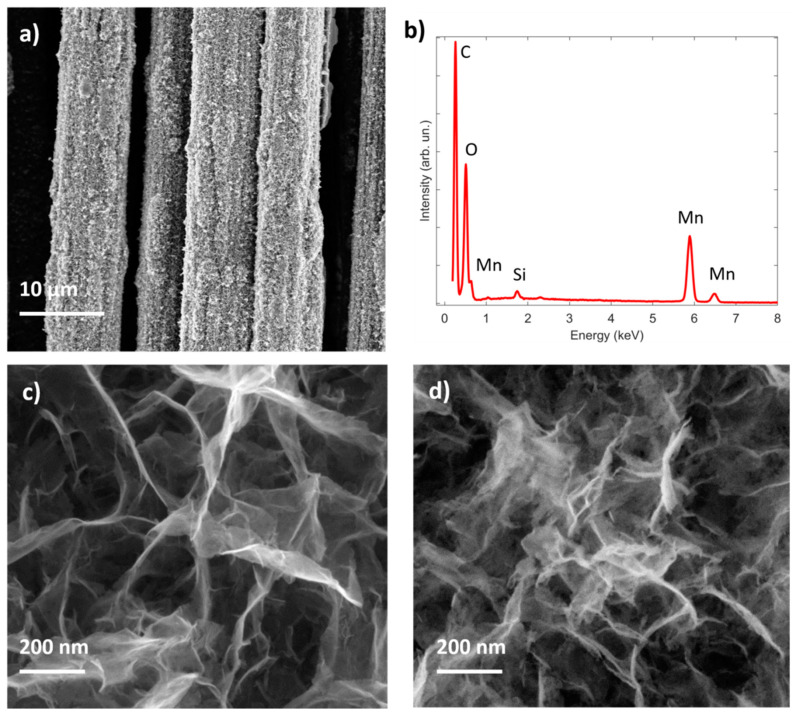
(**a**) Field-Emission Scanning Electron Microscope (FESEM) image of the 2-step electrodeposited manganese oxide showing good coverage of the carbon fibers and (**b**) representative Energy Dispersive X-ray (EDX) spectrum; higher magnification FESEM images of the 2-step electrodeposited manganese oxide before (**c**) and after (**d**) the thermal treatment in H_2_.

**Figure 2:**
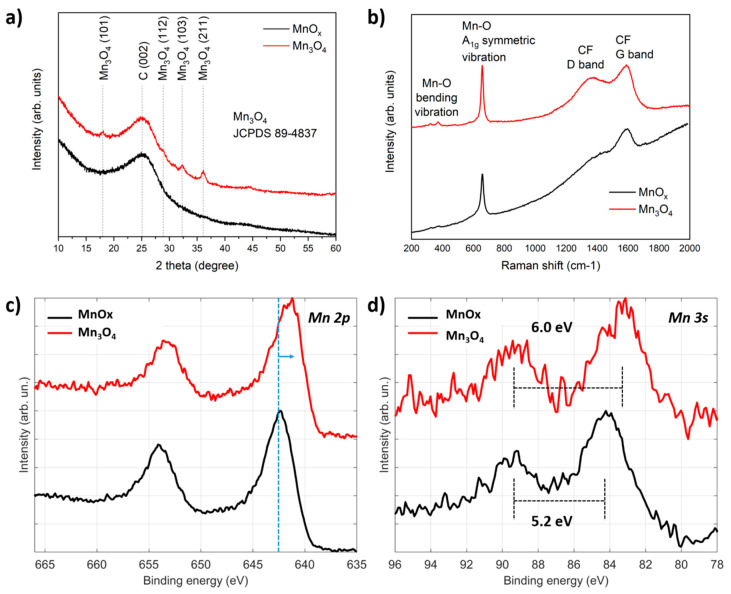
(**a**,**b**) Show the XRD and Raman spectra of MnO_x_ and Mn_3_O_4_, (**c**,**d**) provide XPS spectra of Mn 2p and Mn 3s regions of manganese oxide before (MnOx) and after (Mn_3_O_4_) the thermal treatment in H_2_.

The description of the first fabrication step is reported in [35]; herein, a further improvement is presented, where the MnO_x_ decorated carbon fibers were exposed to a thermal treatment at 250 °C under hydrogen flow for 3.5 h. As shown in Figure 1a, the electrodeposition process does not produce any cracks in the manganese oxide film, and a uniform coverage of nanosheets (Figure 1c) is obtained, in accordance with the previous literature [36,37]. EDX analysis of the electrodeposited manganese oxide electrode (Figure 1b) confirms the expected chemical composition showing C, O and Mn X-ray peaks, with a negligible contribution by Si, which can be ascribed to the internal fluorescence of the Si(Li) detector or surface contamination. It is interesting to notice that, after the thermal treatment, a modification of the surface of the nanosheets is obtained (Figure 1d), resulting in increased roughness and, consequently, an increased surface area of the electrode. This trend is further confirmed by the electrical double layer capacitance value which is proportional to the active surface area (see Figure S1, supporting information). In order to identify a possible phase transformation during this treatment, X-ray diffraction analysis was performed. The thermal treatment under hydrogen flow induces a crystallization of the material to Mn_3_O_4_ in the spinel form (weak signal), as clearly evidenced in the XRD pattern reported in Figure 2a. As reported previously [35], the manganese oxide film is amorphous after the electrodeposition process, while after the hydrogenation, it is possible to identify peaks from the (101), (112), (103) and (201) planes of the Mn_3_O_4_ crystallographic structure, superimposed to the typical diffraction pattern of CFs.

Raman spectroscopy is less sensitive in the precise identification of the crystallographic behavior of this material, since it is well-established [35,36] that MnO_x_ in their different crystalline forms exhibit Raman signals associated to the motion of the oxygen atoms within the MnO_6_ octahedral units in MnO_x_. The spectrum reported in Figure 2b exhibits an intense and sharp peak at 657 cm^−1^, which can be ascribed to A1g mode which corresponds to the Mn–O breathing vibration of Mn^2+^ ions in tetrahedral coordination, and two weak features at 371 and 318 cm^−1^, respectively, related to Mn−O bending vibrations. Such features are more evident after calcination, but are visible also in the pristine material. Thermal process in H_2_ strongly reduces the luminescence emission, related with the growing behavior for high wavenumbers superimposed to the spectrum in pristine material.

A major contribution to unravel the composition of the annealed manganese oxide was given by XPS to study the change in surface composition. In fact, the Mn 2p and Mn 3s regions of the photoelectron spectrum provide useful information for the phase identification of manganese oxide species. Regarding the Mn 2p region (Figure 2c), a shift to lower binding energy of the Mn 2p_3/2_ peak after the hydrogen treatment suggests a decrease in the oxidation state of Mn, in accordance with the literature [37,38,39]. Moreover, the absence of shake-up satellites at the higher binding energy side of the Mn 2p_3/2_ peak excludes the presence of the MnO in significant amounts [40]. In the Mn 3s spectrum (Figure 2d) of the untreated manganese oxide and of the sample after annealing in H_2_ flow, a substantial change in the energy difference of the multiplet split components is shown, due to a change in the oxidation state. The larger the energy difference ∆E of the multiplet split components, the lower the Mn average oxidation state (AOS), according to the formula AOS = 8.95 − 1.13·∆E [41]. The Mn_3_O_4_ sample shows a larger ∆E (6 eV) as compared to (5.2 eV) for the untreated manganese oxide sample, indicating a decrease in AOS from 3.1 (MnO_x_) to 2.2 (Mn_3_O_4_), based on the previously reported formula. In summary, XPS confirms that the H_2_ treatment lowers the average oxidation state of Mn atoms, in accordance with the structural characterization and with the literature [33].

### 3.2. Characterization of the Material at the Anode

**Figure 3:**
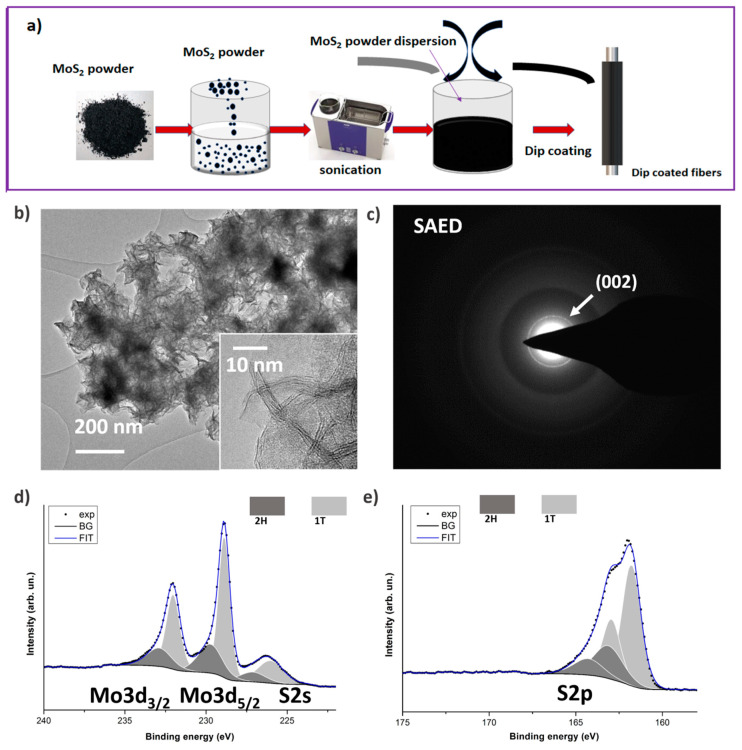
(**a**) Schematic representation of dip-coating of MoS_2_ on carbon fiber (CF); (**b**) shows a low-magnification TEM image of the MoS_2_ nanoflakes, while the inset provides a high-magnification image showing the layered structure of the nanoflakes, (**c**) provides a representative electron diffraction pattern. XPS high-resolution scans of Mo3d/S2s region (**d**) and S2p region (**e**) for MoS_2_ nanoflakes are presented.

Figure 3a shows a schematic representation of the fabrication of the anode electrode, in which MoS_2_ nanoflakes were dip-coated on carbon fiber. The MoS_2_ nanoflakes were hydrothermally synthesized using phosphomolybdic acid and L-Cysteine as precursors in an autoclave at 180 °C for 12 h (details are provided in the experimental section), according to the procedure previously published [24]. Through this particular synthetic approach, it is possible to obtain few-layer nanoflakes with thickness <5 nm and lateral size <100 nm (Figure 3b). Electrodes which exhibit 2D morphology and, in particular, transition metal dichalcogenides (TMD) are well known to be highly efficient for a rapid electron/ion transport [42]. The open structure allows fast variations of the volume, thus increasing the stability of the electrodes upon charge and discharge cycles, even when subjected to intercalation of ions [43]. Based on the electron diffraction measurements (Figure 3c), the nanoflakes exhibit a layered structure (~6.1 Å interplanar spacing (002)), and the absence of in-plane ordering (diffused rings in the diffraction pattern). Further information on the structure is obtained from XPS, where two sets of peaks separated by 0.9 eV in binding energy [23], [44] in the Mo3d/S2s (Figure 3d) and S2p (Figure 3e) regions of the photoelectron spectrum point to the presence of short range domains of 1T and 2H polymorphs with a ratio 1T/2H~2.2. This is particularly interesting for applications of mixed-phase 1T-2H MoS_2_ nanostructures, because the presence of the metallic (1T) phase assures a better electron conductivity than only semi-conductive structures (2H), and this leads to much faster charge transfer kinetics at the nanosheets [45].

### 3.3. Electrochemical Characterization

**Figure 4:**
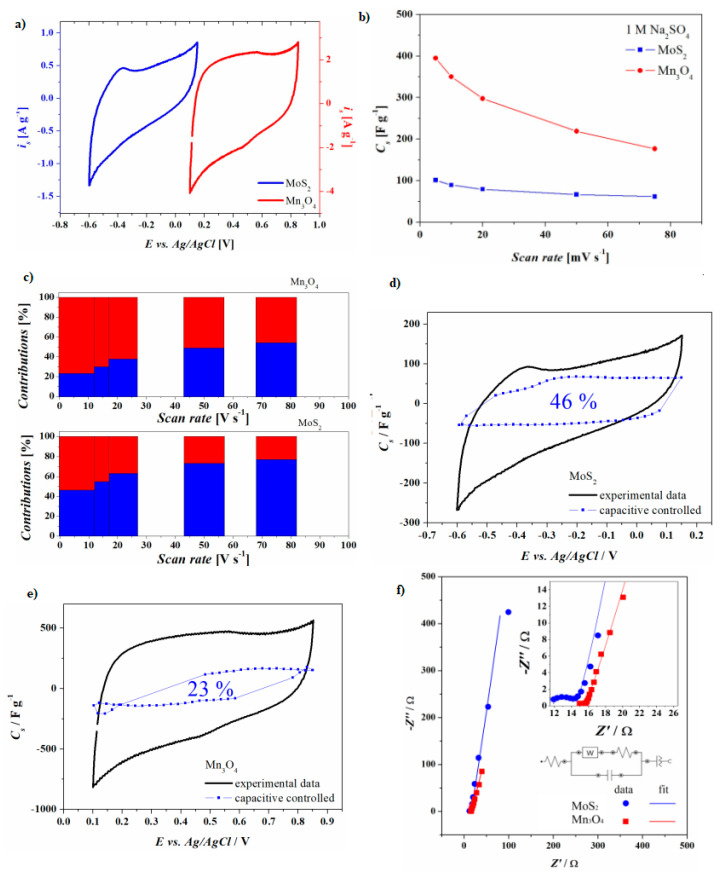
Comparison of cyclic voltammetry (**a**), comparison of specific capacitances of MoS_2_ (blue graphs, 0.85 mg) and Mn_3_O_4_ (red graphs, 0.4 mg) electrodes (**b**). Estimation of the charge stored by diffusion-controlled phenomena (red) and capacitive controlled phenomena (blue) at different scan rates on MoS_2_ (lower graph) and Mn_3_O_4_ (upper graph) electrodes (**c**), comparison of experimental data with computed data for MoS_2_ and Mn_3_O_4_ electrodes, respectively (**d**,**e**), AC impedance spectroscopy and Nyquist plot up to 10 mHz of the MoS_2_ electrode (blue) and Mn_3_O_4_ electrodes red (**f**), inset shows the equivalent circuit of electrode, respectively.

The behavior of MoS_2_ and Mn_3_O_4_ electrodes for supercapacitors was first investigated in 1M Na_2_SO_4_ in a three electrodes configuration, in which the modified fibers were used as the working electrodes and a platinum bar and a saturated Ag/AgCl were employed as counter and reference. In Figure 4a, the cyclic voltammetries at 5 mV s^−1^ of the two samples are shown together, in order to appreciate their compatible voltage windows. In this experiment, the length of the MoS_2_ electrode was increased with respect to the Mn_3_O_4_, in order to equilibrate the charges stored by the two electrodes. The performance of the Mn_3_O_4_ electrode was studied and compared with the behavior of the same sample prior to thermal treatment, and the Mn_3_O_4_ electrode has shown better performance than the latter (see Figure S1, supporting information). Mn_3_O_4_ electrodes were tested between 0.1 V and 0.85 V vs. Ag/AgCl showing quasi-rectangular shapes at all the scan rates employed. The capacitive performances of the MoS_2_ electrode relies in the mixed 1T-2H phases obtained by simple hydrothermal synthesis of the flakes that are nicely attached onto the carbon fibers without any binder [23]. Cyclic voltammograms acquired at different scan rates show a potential window from -0.6 V to + 0.15 V vs. Ag/AgCl. In Figure 4b, the specific capacitance values of the two electrodes are shown. According to this plot, for MoS_2_ electrodes, the capacitance remains almost constant up to 20 mV s^−1^, and for slower scan rates it increases. For the Mn_3_O_4_ samples, the capacitance rises with the decrease of the scan rate, therefore, the total amount of stored charges is strongly time dependent. In order to understand the storage mechanisms of the two different electrodes, the total charge has to be separated in three components: (i) the non-faradaic currents due to the double-layer; (ii) near-surface adsorption giving rise to the pseudocapacitance; and (iii) the faradaic currents rising from charge transfer and diffusion of ions inside the structure; [25,46,47]. As the first two phenomena are capacitive-controlled, with the current proportional to the scan rate (ν) while the latter is diffusion-controlled, the current is proportional to the square root of the scan rate (ν^1/2^). At each potential, the current i(V) can be divided in two contributions that are capacitive controlled (kaν) and diffusion-controlled (kbν^1/2^):

i(V) = k_a_ · ν + k_b_ · ν^1/2^ (1)

This method makes it possible to divide the fraction of current due to capacitive effects (EDLC and pseudocapacitance) and the fraction due to intercalation/insertion of cations in the active material. In the MoS_2_ electrodes, the capacitive controlled mechanism is predominant for scan rates higher than 10 mV s^−1^ (55 %), while in the Mn_3_O_4_ electrodes, it is predominant for scan rates higher than 50 mV s^−1^ (58%). For lower scan rates, the diffusion-controlled current becomes predominant, as clearly seen in the red plots in Figure 4c, where the partition of the charges due to the two phenomena are visualized as a percentage of the total charge. Figure 4d,e shows the experimental voltammetry (black line) with the simulated capacitive-controlled current (blue line and dots), for the MoS_2_ and Mn_3_O_4_ electrodes, respectively. In the simulated curve, the presence of dots indicates a χ^2^ larger than 0.9 in the fitting procedure. The charge-storage mechanism in Mn_3_O_4_ is similar to that of untreated MnO_x_, with Faradic reactions occurring both on the surface and in the bulk of the electrode. The surface Faradaic reaction involves the adsorption/desorption of alkali metal cations (called cat^+^ such as H^+^, Li^+^, Na^+^, K^+^) on the manganese oxide in neutral electrolyte. In both mechanisms of charge-storage, redox reactions between the 2+, 3+, and 4+ oxidation states of Mn ions occur [48]. Differently from the well-known ideal rectangular shape of double-layer capacitors, in Mn_3_O_4_ a pair of peaks are easily distinguished at 0.48 V and 0.56 V, which are rising from the redox transitions of Mn between multiple valence states counterbalanced by the intercalation/deintercalation of cations [49,50] in the spinel 3D-tunnels of the material. The spinel’s open structure, in fact, improves the diffusion inside the Mn_3_O_4_ microstructure [51,52]. In neutral electrolytes, the suggested reaction path and storage mechanisms are [53]:

(2) $$2\left(Mn_{3}O_{4}\cdot 2H_{2}O\right)+5OH^{-}\;\to \;6MnO_{2}\cdot 5H_{2}O+3H^{+}+8e^{-}$$

The efficiency conversion of this reaction step is highly dependent on the preconditioning time (or number of cycles when cyclic voltammetry is employed). Herein, the Mn_3_O_4_ electrodes reached a stable voltammetry after 300 cycles. The reversible reactions are:

(3) $$MnO_{2}{}_{surface}\;\cdot nH_{2}O+cat^{+}+e^{-}\;\begin{array}{c} \to \\ \leftarrow \end{array}\;MnO_{2}^{-}cat^{+}{}_{surface}\cdot nH_{2}O$$

In the previous literature, this mechanism was suggested to be predominant in amorphous MnO_2_, or when the sample presents large specific surface area [47,54], and it was associated with optimal particle size and pore distribution. The second mechanism was suggested [47,55,56] to be predominant for crystalline MnO_2_, in which the crystallographic cell provide tunnels in which limited Faradaic reactions with intercalation/deintercalation of cations in the electrolyte takes place:

(4) $$MnO_{2}\cdot nH_{2}O+cat^{+}+e^{-}\;\begin{array}{c} \to \\ \leftarrow \end{array}\;MnOOcat\cdot nH_{2}O$$

In this particular compound, both charge storage mechanisms (2) and (3) take place: the first due to the open channels of the spinel structure, which offer a large surface area, and the second for deeper thicknesses. According to the time-dependent data analysis previously described, the diffusion-controlled capacitance is significantly high in the sample Mn_3_O_4_ and it dominates also at fast rates (50 mV s^−1^). The main mechanism is therefore the intercalation/deintercalation of cations or protons into/out the Mn_3_O_4_ nanostructures. The quasi-rectangular shape of the voltammetries at all the scan rates indicates a fast charge/discharge process that happens at an almost constant rate, even for solid state diffusions. This may be due to the thermal treatment in hydrogen atmosphere that enhances the surface porosity and generates high surface area with more active Mn (II) and Mn (III) sites for a rapid intercalation and extraction of cations.

In MoS_2_, the quasi-rectangular nature of the CV curves indicates the capacitive behavior of such electrodes. The deviation from a perfect rectangular behavior can be attributed to the electrosorption of protons on the surface of the nanosheets [56]. The adsorption of H^+^ leads, in fact, to the formation of molecular hydrogen and evolution of the gas, not only in acidic media [45,57,58], but also in neutral electrolytes [59]. As discussed above in the XPS and TEM paragraphs, the hydrothermal synthesis employed in this study makes it possible to obtain a mixed 1T-2H phase of MoS_2_, and the electrodes were obtained simply by dip coating the suspension on the carbon fibers with no binder addition. This makes it very challenging to identify a univocal onset potential of the HER from sample to sample and therefore to decide the lower potential limit of the voltammetry. In 2D-MoS_2_ flakes, the nature of the storage mechanisms [59,60] are the formation of the electrical double layer with the adsorption/desorption of ions:

(5) $$MoS_{2_{surface}}+cat^{+}+e^{-}\;\begin{array}{c} \to \\ \leftarrow \end{array}\;MoS_{2}^{-}cat^{+}{}_{surface}$$

and the pseudocapacitance [23,61] with the insertion/extraction of protons and alkali cations between the interlayer spaces:

(6) $$MoS_{2}+cat^{+}+e^{-}\;\begin{array}{c} \to \\ \leftarrow \end{array}\;MoS-Scat$$

Both these two phenomena are capacitive-controlled in nature, which is in fact the main contribution to the total current of MoS_2_ samples (Figure 4c), unless very slow rates of charge and discharge are applied. When very slow voltammetry is performed, different phenomena start to take place and get over. This current can be due to diffusion phenomena that mainly take place on the MoS_2_ and, to some extent, on the carbon fiber substrate that is partially exposed to the solution due to the absence of a binder or a compact/blocking layer, as it has been observed for different carbonaceous materials [61]. AC impedance measurement was carried out at the open circuit potentials of each electrode as shown in Figure 4f. From the data fitting, it is possible to appreciate the uncompensated resistance (highest frequencies) characteristic of each material-interfaces, the constant phase element (lower frequencies) that mimic imperfect capacitor (EDLC and pseudocapacitance) and the Randles’ circuit in which the Warburg impedance (medium frequencies) describes the diffusion-controlled phenomena previously analyzed and commented in the text. The uncompensated resistance is 14.6 Ω and 11.9 Ω for the Mn_3_O_4_ and MoS_2_ electrodes, respectively, while the charge transfer resistance values of 0.9 Ω and 2.5 Ω suggest fast electron transfer in both materials.

**Figure 5:**
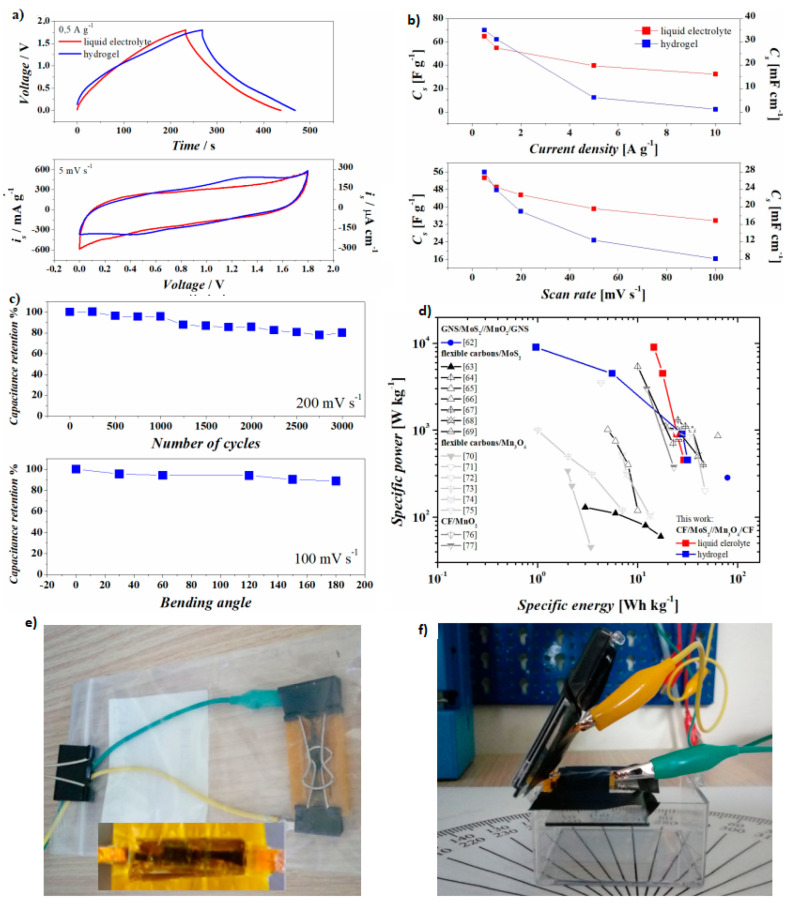
Electrochemical experiments on flexible devices employing liquid electrolytes (blue) and hydrogel (red): (**a**) charge and discharge at 0.5 A g^−1^ (upper) and cyclic voltammetry at 5 mV s^−1^ (lower), (**b**) specific capacitance estimated on charge and discharge experiments (upper) and on cyclic voltammetry (lower), (**c**) cyclability and bending stability, (**d**) Ragone plot and the pictures of the device with hydrogel during ageing (inset shows the device) (**e**) and bending (**f**) tests.

After the study of the single electrodes, the MoS_2_ electrode was employed as an anode and the Mn_3_O_4_ was placed as a cathode in asymmetric supercapacitors. With this intent, two configurations of asymmetric supercapacitors were chosen: (i) a standard asymmetric device by using 1M Na_2_SO_4_ electrolyte (called liquid electrolyte) and (ii) an asymmetric device by using PVA based gel (called hydrogel) in order to make the cell appealing for commercial flexible device with large bending stability over multiple angles. Figure 5a shows the comparison of charge-discharge profiles (upper graph) and CV (lower graph) of the devices in liquid electrolyte and in hydrogel electrolyte. The two methods show consistent behaviors at multiple current densities and scan rates: as shown in Figure 5b, the specific capacitance estimated from the galvanostatic experiments and from cyclic voltammetries show that the devices made with hydrogel have larger capacitance at low rates, while the liquid electrolyte devices provide larger capacitance even at faster rates. Decreasing the rates of the experiments, the capacitance values of the two devices merge to quite the same value (i.e., the specific capacitances at 0.5 A g^−1^ are 65 F g^−1^ and 70 F g^−1^). Wearable and flexible devices are those in which the performances of the devices remain almost invariant when the electrodes are bended, folded and wrapped. The presence of liquid electrolyte soaked in a membrane is a major limitation on the flexibility, because by bending the device (i) the electrolyte moves and this creates inhomogeneity at the electrode-electrolyte interface and (ii) the roughness of the membrane scratches the active material away from the fibers. With this aim, the devices with hydrogel were aged (shown in Figure 5c bottom). From the bending experiments, it was observed that the capacitance decreased of 12% with respect to the initial value, when the device was completely folded (180°). The devices with hydrogel were cycled at 200 mV s^−1^ up to 3000 scans, showing a capacitance retention of 80%, which could be increased indeed by improving the packaging and sealing. The performances of the two devices are compared in Figure 5d, with the literature on supercapacitors in which MoS_2_ and MnO_2_ were employed on steady Ni-foam current collectors and cellulose separator soaked of hydrogel [62], bendable supercapacitors in which one of the electrodes includes a flexible carbon-based current collector decorated with MoS_2_ [63,64,65,66,67,68,69] with Mn_3_O_4_ [70,71,72,73,74,75]_,_ or specifically with carbon fibers coated with MnO_2_ [76,77]. In particular, at powers from 9000 to 450 W/kg, the device in this study based on liquid electrolyte delivers from 14 to 29 Wh/kg, while that with hydrogel delivers 1 to 31 Wh/kg.

## 4. Conclusions

Mn_3_O_4_ on carbon fibers has been fabricated by anodic electrodeposition and annealing in reducing atmosphere and used as flexible cathode. Two-dimensional flakes of mixed phase 1T-2H MoS_2_ were synthetized by hydrothermal method and they were employed as binder-free active material on carbon fibers as flexible anode. Asymmetric supercapacitors assembled with neutral electrolyte deliver at 0.5 A g^−1^ a device capacitance of 65 F g^−1^ (32 mF cm^−1^), and when the gel electrolyte is employed, it increases up to 70 F g^−1^ (35 mF cm^−1^). The latter layout makes it possible not only to achieve higher level of capacitance, but also to use such device under bending conditions with limited capacitance fading also after 3000 cycles. The present results demonstrate that these flexible hybrid devices which involve all the electrochemical storage mechanisms can increase the typical energy range and maintain the same speed (power) of supercapacitors.

## Supplementary Materials

The following are available online at https://www.mdpi.com/2079-4991/10/6/1084/s1, Figure S1: (a,b) cyclic voltammetries recorded at different scan rates for both MnO_x_ and Mn_3_O_4_ (before and after thermal treatment, respectivelly), (c,d) cyclic voltammetries and capacitance comparison for MnO_x_ and Mn_3_O_4_, Table S1: comparison of voltage windows and specific capacitances.
