# Supplementary material for: Binder Free and Flexible Asymmetric Supercapacitor Exploiting Mn3O4 and MoS2 Nanoflakes on Carbon Fibers
Source: Nanomaterials (Basel). 2020 May 31;10(6):1084. doi: 10.3390/nano10061084 (PMC7353199; doi:10.3390/nano10061084)
Supplement: Supplementary file 1 [file nanomaterials-10-01084-s001.pdf]

Supporting Information

Binder Free and Flexible Asymmetric Supercapacitor Exploiting Mn_3O_4 and MoS_2 Nanoflakes on Carbon Fibers

Amjid Rafique [1,*], Usman Zubair [1,2], Mara Serrapede [3], Marco Fontana [3], Stefano Bianco [1], Paola Rivolo [1], Candido F. Pirri [1,3] and Andrea Lamberti [1,3,*]

[1] Politecnico di Torino, Dipartimento di Scienza Applicata e Tecnologia (DISAT), Corso Duca Degli Abruzzi, 24, 10129 Turin, Italy; usman.zubair@polito.it (U.Z.); stefano.bianco@polito.it (S.B.); paola.rivolo@polito.it (P.R.); fabrizio.pirri@polito.it (C.F.P.)

[2] Faculty of Engineering and Technology, National Textile University, Faisalabad 38000, Pakistan

[3] Istituto Italiano di Tecnologia, Center for Sustainable Future Technologies, Corso Trento, 21, 10129 Turin, Italy; mara.serrapede@iit.it (M.S.); marco.fontana@iit.it (M.F.)

* Correspondence: amjid.rafique@polito.it (A.R.); andrea.lamberti@polito.it (A.L.)

Table S1. comparison of Voltage windows and specific capacitances.

Electrode composition	electrolyte	Voltage window (V)	Specific Capacitance ($F\ g^{-1}$)	Energy density $W\ h\ kg^{-1}$	Power density $kW\ kg^{-1}$	Ref
**Graphene/MnO_2//Graphene**	1M Na_2SO_4	1.7	25.5	10.03	2.53	S1
**AC// MnO_2**	1M Na_2SO_4	1.8	23.1	10.4	14.7	S2
**AC// $NaMnO_2$**	1M Na_2SO_4	1.9	38	19.5	0.13	S3
**VO_2/ rGO// Mn_3O_4/rGO**	1M Na_2SO_4	2.2	90	42.7	1.1	S4
**AC//Mn_3O_4/Graphite paper**	1MNa_2SO_4	1.6	91	47	0.202	S5
**MnO_2 Nanosheet// Carbon Fiber**	PVA/LiCL	1.5	87.1	27.7		S6
**MnO_2/GNS//MoS_2 /GNS**	PVA/Na_2SO_4 (1M Na_2SO_4)	2.0	142	78.9	0.248	S7
**Fe_2O_3/Carbon fabric// MnO_2/Carbon fabric**	PVA/LiCl (1M LiCl)	1.8	119	53		S8
**MoS_2/CF//Mn_3O_4/CF**	1M Na_2SO_4	1.8	70	30	9	This work
**MoS_2/CF//Mn_3O_4/CF**	PVA/Na_2SO_4 (1M Na_2SO_4)	1.8	65	29	0.45	This work

The above table S1 give the comparison of our devices with literature to show that we have the comparable results. The data shows, we succeed in getting high voltage of 1.8 V with comparable specific capacitance.

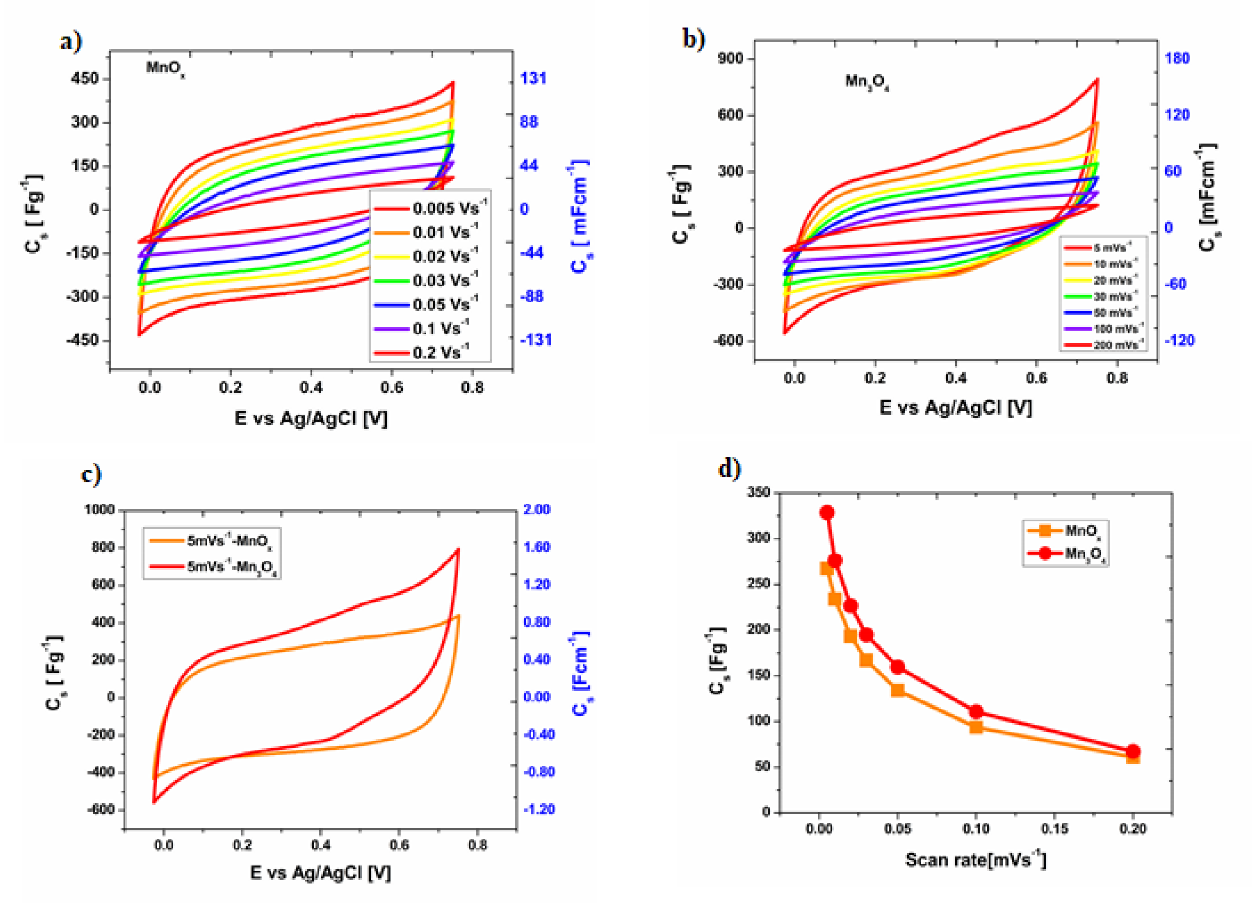

Figure S1. a–b CV's recorded at different scan rate for both MnO_x and Mn_3O_4 (before and after thermal treatment, c-d) show the CV and capacitance comparison for MnO_x and Mn_3O_4.

References

S1 L. Deng, G. Zhu, J. Wang, L. Kang, Z.-H. Liu, Z. Yang, Z. Wang, Graphene–MnO_2 and graphene asymmetrical electrochemical capacitor with a high energy density in aqueous electrolyte, Journal of Power Sources, 2011, 196, 10782-10787

S2 Y.-T. Wang, A.-H. Lu, H.-L. Zhang, and W.-C. Li, Synthesis of Nanostructured Mesoporous Manganese Oxides with Three-Dimensional Frameworks and Their Application in Supercapacitors, J. of Phy. Chem. C, 2011,115, 5413-5421

S3 Q.T.Qu, Y. Shi, S.Tian , Y.H. Chen, Y.P. Wu, R. Holze, A new cheap asymmetric aqueous supercapacitor: Activated carbon//NaMnO2, Journal of Power Sources, 194, 2009, 1222–1225

S4 R. Sahoo, D. T. Pham, T. H. Lee, T. H. T. Luu, J. Seok, and Y. H. Lee, Redox-Driven Route for Widening Voltage Window in Asymmetric Supercapacitor, ACS Nano, 2018, 12, 8, 8494-8505.

S5 Aswathy, M. Ulaganathan, P. Ragupathy, Mn_3O_4 nanoparticles grown on surface activated graphite paper for,aqueous asymmetric supercapacitors, Journal of Alloys and Compounds, 2018, 767, 141-150.

S6 N. Yu, H. Yin, W. Zhang, Y. Liu, Z. Tang, M.-Q. Zhu, High-Performance Fiber-Shaped All-Solid-State Asymmetric Supercapacitors Based on Ultrathin MnO_2 Nanosheet/Carbon Fiber Cathodes for Wearable Electronics, Adv. Energy Mater., 2016, 6, 1501458.

S7 X. Yang, H. Niu, H. Jiang, Q. Wang, and F. Qu, A high energy density all-solid-state asymmetric supercapacitor based on MoS_2/graphene nanosheets and MnO_2/graphene hybrid electrodes. Mater. Chem. A, 2016, 4, 11264-11275.

S8 W. Liu, M. Zhu, J. Liu, X. Li, J. Liu, Flexible asymmetric supercapacitor with high energy density based on optimized MnO_2 cathode and Fe_2O_3 anode, Chinese Chemical Letter, 2019, 750-756
